# Carbon-Ion Beam Irradiation Kills X-Ray-Resistant p53-Null Cancer Cells by Inducing Mitotic Catastrophe

**DOI:** 10.1371/journal.pone.0115121

**Published:** 2014-12-22

**Authors:** Napapat Amornwichet, Takahiro Oike, Atsushi Shibata, Hideaki Ogiwara, Naoto Tsuchiya, Motohiro Yamauchi, Yuka Saitoh, Ryota Sekine, Mayu Isono, Yukari Yoshida, Tatsuya Ohno, Takashi Kohno, Takashi Nakano

**Affiliations:** 1 Department of Radiation Oncology, Gunma University Graduate School of Medicine, Maebashi, Gunma, Japan; 2 Department of Radiology, Chulalongkorn University, Pathumwan, Bangkok, Thailand; 3 Division of Genome Biology, National Cancer Center Research Institute, Chuo-ku, Tokyo, Japan; 4 Advanced Scientific Research Leaders Development Unit, Gunma University, Maebashi, Gunma, Japan; 5 Division of Radiation Biology and Protection, Atomic Bomb Disease Institute, Nagasaki University, Sakamoto, Nagasaki, Japan; 6 Gunma University Heavy Ion Medical Center, Maebashi, Gunma, Japan; University of Hawaii Cancer Center, United States of America

## Abstract

**Background and Purpose:**

To understand the mechanisms involved in the strong killing effect of carbon-ion beam irradiation on cancer cells with *TP53* tumor suppressor gene deficiencies.

**Materials and Methods:**

DNA damage responses after carbon-ion beam or X-ray irradiation in isogenic HCT116 colorectal cancer cell lines with and without *TP53* (p53^+/+^ and p53^-/-^, respectively) were analyzed as follows: cell survival by clonogenic assay, cell death modes by morphologic observation of DAPI-stained nuclei, DNA double-strand breaks (DSBs) by immunostaining of phosphorylated H2AX (γH2AX), and cell cycle by flow cytometry and immunostaining of Ser10-phosphorylated histone H3.

**Results:**

The p53^-/-^ cells were more resistant than the p53^+/+^ cells to X-ray irradiation, while the sensitivities of the p53^+/+^ and p53^-/-^ cells to carbon-ion beam irradiation were comparable. X-ray and carbon-ion beam irradiations predominantly induced apoptosis of the p53^+/+^ cells but not the p53^-/-^ cells. In the p53^-/-^ cells, carbon-ion beam irradiation, but not X-ray irradiation, markedly induced mitotic catastrophe that was associated with premature mitotic entry with harboring long-retained DSBs at 24 h post-irradiation.

**Conclusions:**

Efficient induction of mitotic catastrophe in apoptosis-resistant p53-deficient cells implies a strong cancer cell-killing effect of carbon-ion beam irradiation that is independent of the p53 status, suggesting its biological advantage over X-ray treatment.

## Introduction

Carbon-ion radiotherapy has been provoking interest in the field of cancer therapy. Carbon-ion beams have advantageous properties over X-ray; a superior dose distribution associated with the sharp penumbra and the Bragg peak, and strong cell-killing effect [1], [2]. The major promising clinical outcome of carbon-ion radiotherapy is to overcome the therapeutic resistance of cancer cells to X-ray radiotherapy. For example, a recent study in which carbon-ion radiotherapy was used to treat patients with rectal cancer reported a 5-year local control and overall survival rates of 97% and 51% for post-operative recurrent cases [3]. This rate is superior to the 5-year overall survival rates (0−40%) that are typically achieved by conventional X-ray radiotherapy or surgical resection [3], [4]. However, the biological basis for the strong cell-killing effect of carbon-ion beam irradiation on X-ray-resistant tumors has not been elucidated fully.

Genetic aberrations contribute to the X-ray resistance of cancer cells [5], [6]. Inactivating mutations in the tumor suppressor gene *TP53* are representative of tumor resistance, and these aberrations are associated with poor prognosis after X-ray radiotherapy [7], [8]. The p53 protein plays multiple roles in the DNA damage response (DDR) to X-ray irradiation, including the regulation of cell death pathways and cell cycle checkpoints [9]. The induction of apoptosis by p53 is a key factor affecting the sensitivity of cancer cells to X-ray radiation. Several pre-clinical and clinical studies have demonstrated that *TP53* mutations are associated with the resistance of cancer cells to X-ray irradiation therapy [7], [10], [11].

Previous studies showed that carbon-ion beam irradiation effectively kills X-ray-resistant p53-mutant cancer cells [12––15]. Although the mechanisms involved in this process were examined in these studies, the results were inconsistent. The inconsistencies are likely attributable to the fact that each study focused on only a few aspects of the DDR (such as apoptosis or the cell cycle response) [12]–[15] and each used cancer cell lines with different genetic backgrounds; hence, the effects of aberrations in genes other than *TP53* may have masked the results [12], [13]. Here, to clarify the mechanisms underlying the strong killing effect of carbon-ion beam irradiation on X-ray irradiation-resistant cancer cells with *TP53* aberrations, we performed a comprehensive study of multiple aspects of the DDR using a set of isogenic human cancer cells that differed only in their p53 status.

## Materials and Methods

### Cell lines

Human colorectal cancer HCT116 cells harboring wild-type p53 (p53^+/+^) and its isogenic p53-null derivative (p53^-/-^) were provided by Dr. B. Vogelstein of Johns Hopkins University. HCT116 p53^+/+^ cells have intact DNA damage checkpoints [16]. p53 expression, and the effects of X-ray and carbon-ion beam irradiation on p53 expression in p53^+/+^ and p53^-/-^ cells, was examined by immunoblotting with antibodies against p53 (Santa Cruz) and β-actin (loading control, Cell Signaling Technology) (**S1a Fig.**). There was no significant difference in the population doubling time between the two cell lines (**S1b Fig.**).

Human colon cancer (RKO, LS123, and WiDr) cells, human lung cancer (H1299) cells, and human osteosarcoma (Saos-2) cells were purchased from ATCC. RKO cells harbor wild-type p53. LS123 and WiDr cells harbor a missense mutation in p53 at R175H and R273H, respectively. H1299 and Saos-2 cells are p53-null. H1299 cells stably expressing a p53 missense mutation (R175H, R273H, R249S or R280K) were established as described previously [17]. All cells were cultured in RPMI-1640 medium supplemented with 10% fetal bovine serum.

hTERT-immortalized normal human diploid foreskin fibroblasts (BJ-hTERT) harboring wild-type p53 were purchased from Clontech. BJ-hTERT cells expressing shRNA against EGFP (BJ-hTERT-WT; control) or p53 (BJ-hTERT-shp53) were established as previously described [18], and cultured in Minimum Essential Eagle's Medium.

### Irradiation

X-ray irradiation was performed using a Faxitron RX-650 radiation source (100 kVp, 1.14 Gy/min; Faxitron Bioptics). Carbon-ion beam irradiation was performed at Gunma University Heavy Ion Medical Center using the same beam specifications that are used in clinical settings (290 MeV/nucleon and an average linear energy transfer (LET) at the center of a 6 cm spread-out Bragg peak of approximately 50 keV/µm). Carbon-ion beams were delivered in a vertical direction so that cells on culture plates can receive the dose evenly.

### Clonogenic survival assay

Cells were seeded into 6-well plates and exposed (or not) to X-ray or carbon-ion beam irradiation. After incubation for a further 10 days, the cells were fixed with methanol and stained with crystal violet. Colonies of at least 50 cells were counted. The surviving fraction was normalized to the corresponding controls. The dose that resulted in a surviving fraction of 10% (D_10_) was calculated using the linear-quadratic model, as described previously [19].

### Cell death evaluations

Cells were grown on glass coverslips, exposed (or not) to X-ray or carbon-ion beam irradiation, and then stained with 4',6-diamidino-2-phenylindole dihydrochloride (DAPI), as described previously [20]. Confocal images were collected using a BX51 microscope (Olympus) equipped with a CCD camera (VB-7000; Keyence). Apoptosis was determined based on the morphology of the nuclei, including the presence of apoptotic bodies, nuclear condensation and fragmentation [21]. Cells containing nuclei with two or more distinct lobes were scored as positive for mitotic catastrophe [20], [22]. Cells containing nuclei showing senescence-associated heterochromatic foci were scored as positive for senescence [23]. The percentages of cells undergoing apoptosis, mitotic catastrophe or senescence were quantified by counting at least 300 cells for each experimental condition.

### Cell cycle analysis

Cells exposed (or not) to X-ray or carbon-ion beam irradiation were harvested at the indicated time points, fixed with ethanol, stained with propidium iodide in the presence of RNase, and then analyzed using flow cytometry, as described previously [19].

### Immunostaining

Cells exposed (or not) to X-ray or carbon-ion beam irradiation were stained with antibodies against Ser139-phosphorylated histone H2AX (γH2AX; Millipore) or Ser10-phosphorylated histone H3 (pH3; Millipore), as described previously [24]. γH2AX foci per nucleus were scored in sequential 2D images captured from multiple focal planes. At least 500 cells were evaluated for each experimental condition.

### Statistical analysis

Experiments were performed in triplicate at least unless otherwise stated. Statistically significant differences were determined by unpaired Student's *t*-tests using StatMateIII ver. 3.17 software (ATMS). *P*<0.05 was considered significant.

## Results

### Carbon-ion beams have more potent cancer cell-killing activity than X-rays irrespective of the p53 status

**Figure 1:**
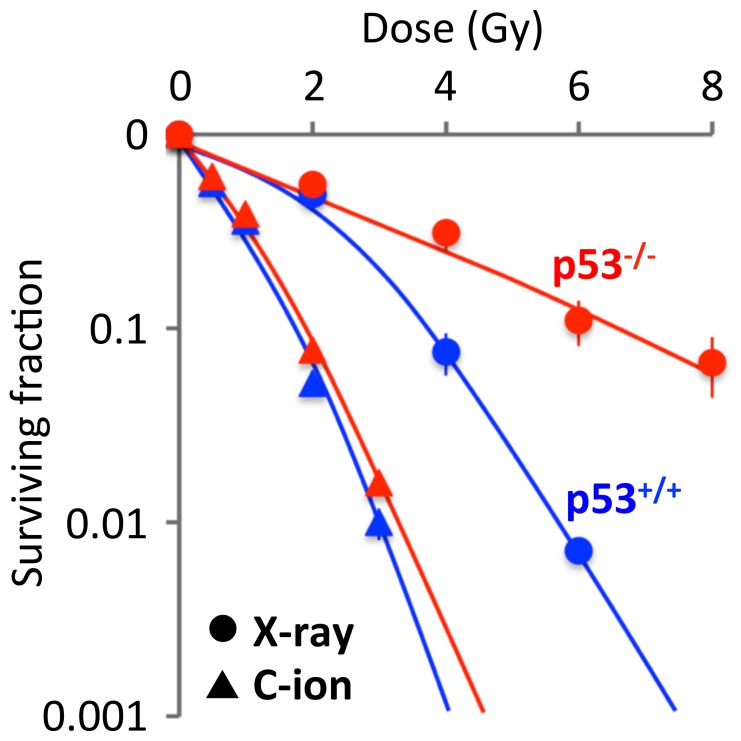
Sensitivity of p53^+/+^ and p53^-/-^ HCT116 cells to X-ray and carbon-ion beam irradiation as assessed by clonogenic survival assays. Cells were seeded in 6-well plates, incubated overnight, and then exposed to X-ray or carbon-ion beam irradiation. After incubation for a further 10 days, the cells were fixed, stained, and counted. The surviving fraction was normalized to the value of the corresponding controls. Data are expressed as the mean ± SD. C-ion, carbon-ion.

The sensitivities of p53^+/+^ and p53^-/-^ HCT116 cells to X-ray and carbon-ion beam irradiation were assessed by clonogenic survival assays (**Fig. 1**). As expected based on the results of previous studies [14], [15], p53^-/-^ cells were more resistant to X-ray irradiation than p53^+/+^ cells; the D_10_ values for these two cell lines were 6.8 Gy and 3.8 Gy, respectively. By contrast, the sensitivities of p53^+/+^ and p53^-/-^ cells to carbon-ion beam irradiation were comparable; the D_10_ values for these cell lines were 1.7 Gy and 1.9 Gy, respectively. Hence, the relative biological effectiveness of carbon-ion beam irradiation to X-ray irradiation at D_10_ was 2.2 in p53^+/+^ cells and 3.6 in p53^-/-^ cells. These data indicate that carbon-ion beam irradiation effectively kills X-ray-resistant *p53*-null cancer cells.

### Aberrations in p53 switch the mode of irradiation-induced cancer cell death from apoptosis to mitotic catastrophe

**Figure 2:**
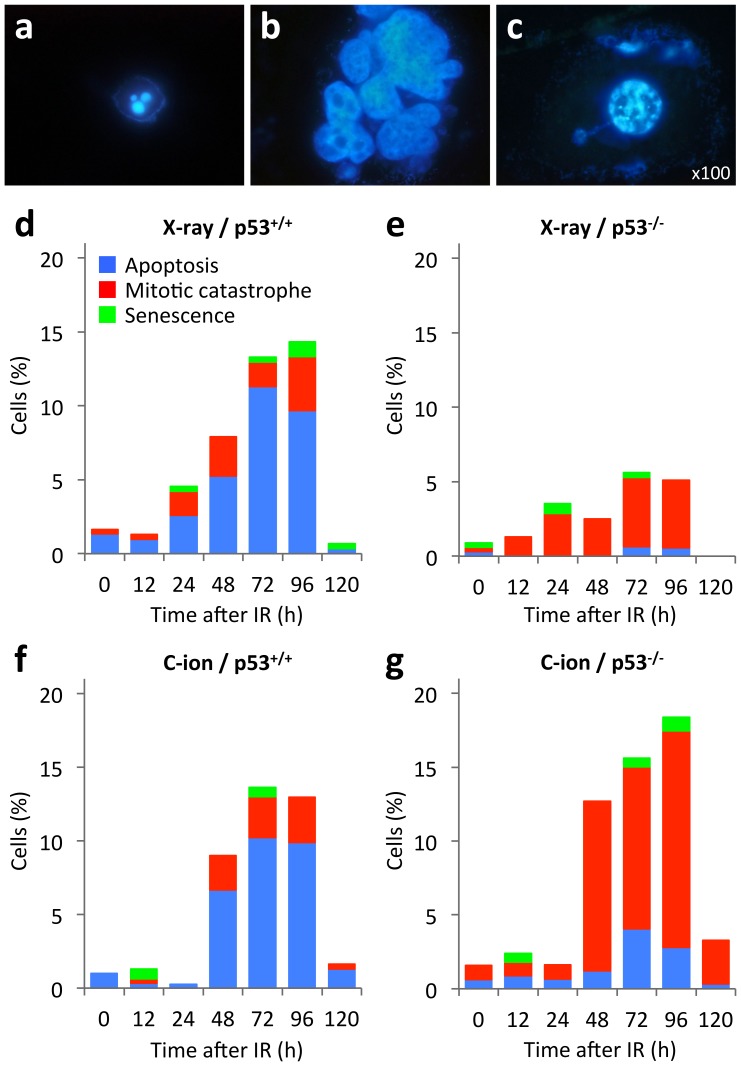
Mode of cell death induced by X-ray or carbon-ion beam irradiation in p53^+/+^ and p53^-/-^ HCT116 cells. Cells seeded on glass coverslips were incubated overnight, exposed (or not; 0 h) to X-ray (4 Gy) or carbon-ion beam (1.5 Gy) irradiation, and then stained with DAPI. Apoptosis, mitotic catastrophe, and senescence were determined according to the characteristic nuclear morphologies (see “Materials and methods” for the definitions). (**a–c**) Representative images showing the nuclear morphology of cells undergoing apoptosis (a), mitotic catastrophe (b), or senescence (c). The images of p53^-/-^ cells were taken 72 h after carbon-ion beam irradiation. (**d, e**) Mode of cell death in p53^+/+^ (d) and p53^-/-^ (e) cells at 0, 12, 24, 48, 72, 96 and 120 h after X-ray irradiation. (**f, g**) Mode of cell death in p53^+/+^ (f) and p53^-/-^ (g) cells at 0, 12, 24, 48, 72, 96 and 120 h after carbon-ion beam irradiation. IR, irradiation; C-ion, carbon-ion.

To explore the mechanisms underlying the p53 status-independent cell-killing activity of carbon-ion beam irradiation, the modes of cell death induced by X-ray or carbon-ion beam irradiation were assessed (**Figs. 2** **,** **3**). p53^+/+^ and p53^-/-^ cells were irradiated with doses of X-ray or carbon-ion beams that were similar to the D_10_ for p53^+/+^ cells (X-ray, 4 Gy; carbon-ion beams, 1.5 Gy). Apoptosis, mitotic catastrophe and senescence were determined by examining the characteristic morphologies of nuclei stained with DAPI (**Fig. 2a–c**) [20]–[23]. In p53^+/+^ cells, apoptosis was the dominant mode of cell death induced by X-ray and carbon-ion beam irradiation (**Figs. 2d, f** **,** **3a**). By contrast, p53^-/-^ cells were less susceptible to apoptosis caused by both types of irradiation (**Figs. 2e, g** **,** **3b**). Interestingly, in p53^-/-^ cells, carbon-ion beam irradiation induced mitotic catastrophe more evidently than X-ray irradiation (**Figs. 2g** **,** **3b**). A higher dose of X-ray irradiation equivalent to the D_10_ (6.8 Gy) for p53^-/-^ cells induced a similar level of mitotic catastrophe to that induced by carbon-ion beam irradiation at 1.5 Gy (**S2 Fig** **.**). The induction of senescence was not evident in all experimental conditions (**Fig. 2**). This result was confirmed by senescence-associated β-galactosidase staining assays, in which the fraction of staining-positive cells was less than 2% for both cell lines exposed to X-ray or carbon-ion beam irradiation (data not shown). These data indicated that apoptosis and mitotic catastrophe is the major mode of cell death in p53^+/+^ cells and p53^-/-^ cells, respectively, both after exposure to X-ray and carbon-ion beam irradiation, and that carbon-ion beam irradiation induces mitotic catastrophe more effectively than X-ray irradiation in apoptosis-resistant p53^-/-^ cells.

**Figure 3:**
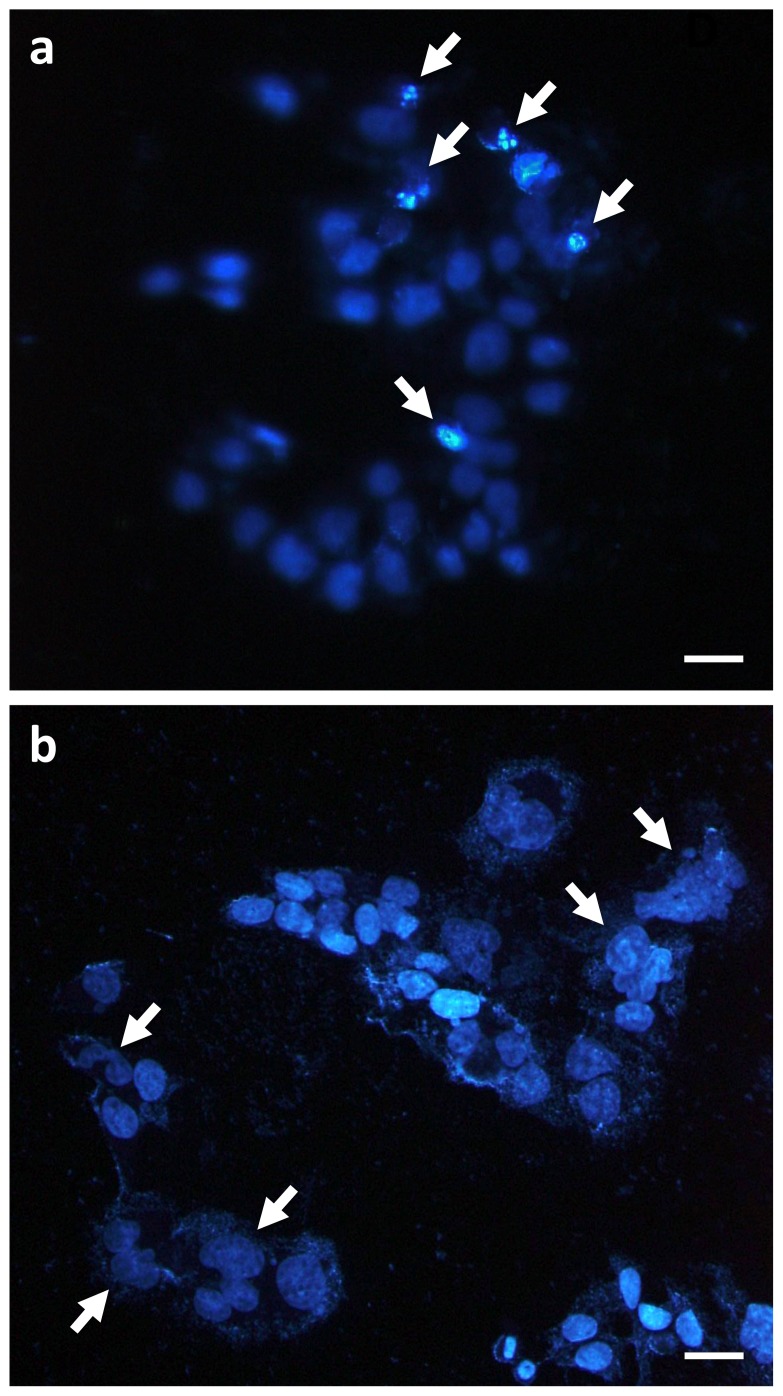
Representative images of p53^+/+^ and p53^-/-^ HCT116 cells irradiated with carbon-ion beams. Cells were seeded on glass coverslips, incubated overnight, exposed to carbon-ion beams (1.5 Gy), and then stained with DAPI 72 h later. Apoptosis, mitotic catastrophe, and senescence were determined according to the characteristic nuclear morphologies (see “Materials and methods” for the definitions). (**a**) p53^+/+^ cells: 12.5%, 0% and 0% of cells showed apoptosis, mitotic catastrophe, and senescence, respectively. (**b**) p53^-/-^ cells: 0%, 12.8% and 0% of cells showed apoptosis, mitotic catastrophe, and senescence, respectively. The arrows in (a) and (b) indicate cells undergoing apoptosis and mitotic catastrophe, respectively. Scale bars, 10 µm.

**Figure 4:**
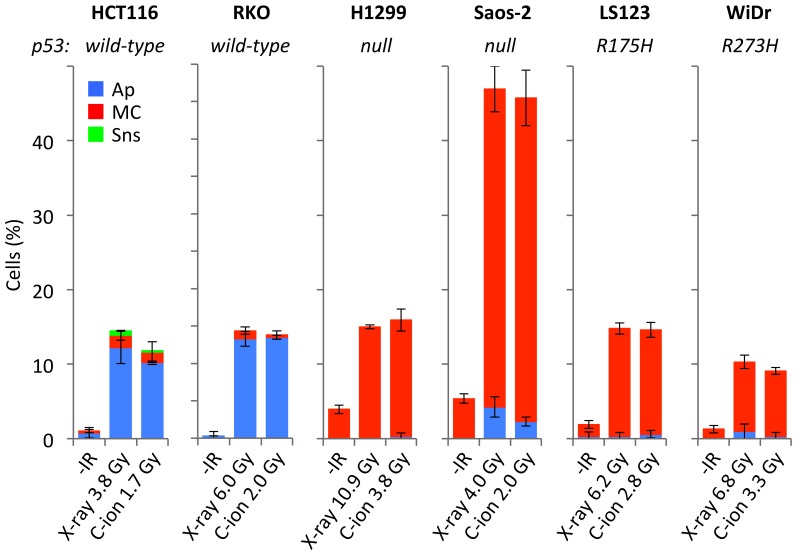
Mode of cell death induced by X-ray or carbon-ion beam irradiation in cancer cell lines with differing *p53* status. Cells were seeded on glass coverslips, incubated overnight, irradiated with X-rays (D_10_ dose) or carbon-ion beams (D_10_ dose), and then stained with DAPI 72 h later. Apoptosis, mitotic catastrophe, and senescence were determined according to the characteristic nuclear morphologies (see “Materials and methods” for the definitions). Data are expressed as the mean ± SD. Ap, apoptosis; MC, mitotic catastrophe; Sns, senescence; IR, irradiation; C-ion, carbon-ion.

**Figure 5:**
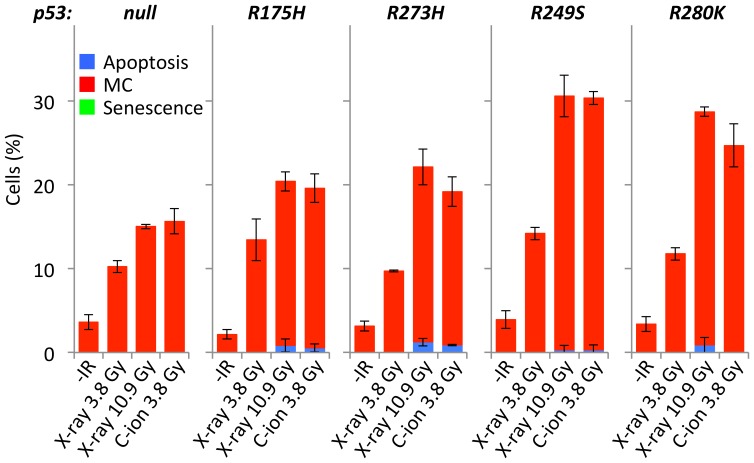
Mode of cell death induced by X-ray or carbon-ion beam irradiation in isogenic H1299 cells expressing different p53 missense mutations. Cells were seeded on glass coverslips, incubated overnight, irradiated with X-rays (10.9 Gy, D_10_ for X-rays; or 3.8 Gy, D_10_ for carbon-ion beams) or carbon-ion beams (3.8 Gy, D_10_ for carbon-ion beams), and then stained with DAPI 72 h later. Apoptosis, mitotic catastrophe, and senescence were determined according to the characteristic nuclear morphologies (see “Materials and methods” for the definitions). Data are expressed as the mean ± SD. MC, mitotic catastrophe; C-ion, carbon-ion; IR, irradiation. Note that a part of p53-null H1299 panel is the same as that shown in Fig. 4 (but the context is now different).

To investigate this further, we examined the mode of cell death in multiple human cell lines with differing p53 status after X-ray or carbon-ion beam irradiation (**Fig. 4**). RKO cells harboring wild-type p53 showed an apoptosis-dominant phenotype after either X-ray or carbon-ion beam irradiation, whereas p53-null H1299 and Saos-2 cells showed a mitotic catastrophe-dominant phenotype. Accordingly, suppression of p53 expression in BJ-hTERT fibroblasts promoted the induction of mitotic catastrophe upon X-ray or carbon-ion beam irradiation (**S3 Fig** **.**). Interestingly, LS123 and WiDr cells (expressing p53 harboring a missense at R175H and R273H, respectively), also showed a mitotic catastrophe-dominant phenotype (**Fig. 4**). These mutation sites are located within the DNA-binding domain of the p53 protein, which plays a key role in the transcriptional activation of several target genes, including those involved in apoptosis induction [25]. Therefore, we next examined the mode of irradiation-induced cell death using a series of isogenic H1299 cells stably expressing p53 proteins harboring missense mutations in the DNA-binding domain that are often observed in human cancers (i.e., R175H, R273H, R249S and R280K) [25]. All of these cell lines showed a mitotic catastrophe-dominant phenotype upon irradiation (**Fig. 5**). Taken together, these results indicate that dysfunction of the p53 DNA-binding domain switches the mode of irradiation-induced cancer cell death from apoptosis to mitotic catastrophe. These results also confirmed that carbon-ion beam irradiation was better than X-ray irradiation at inducing mitotic catastrophe in cancer cells harboring aberrant p53.

### Cells are released from radiation-induced G2/M arrest 24 h after X-ray or carbon-ion beam irradiation

**Figure 6:**
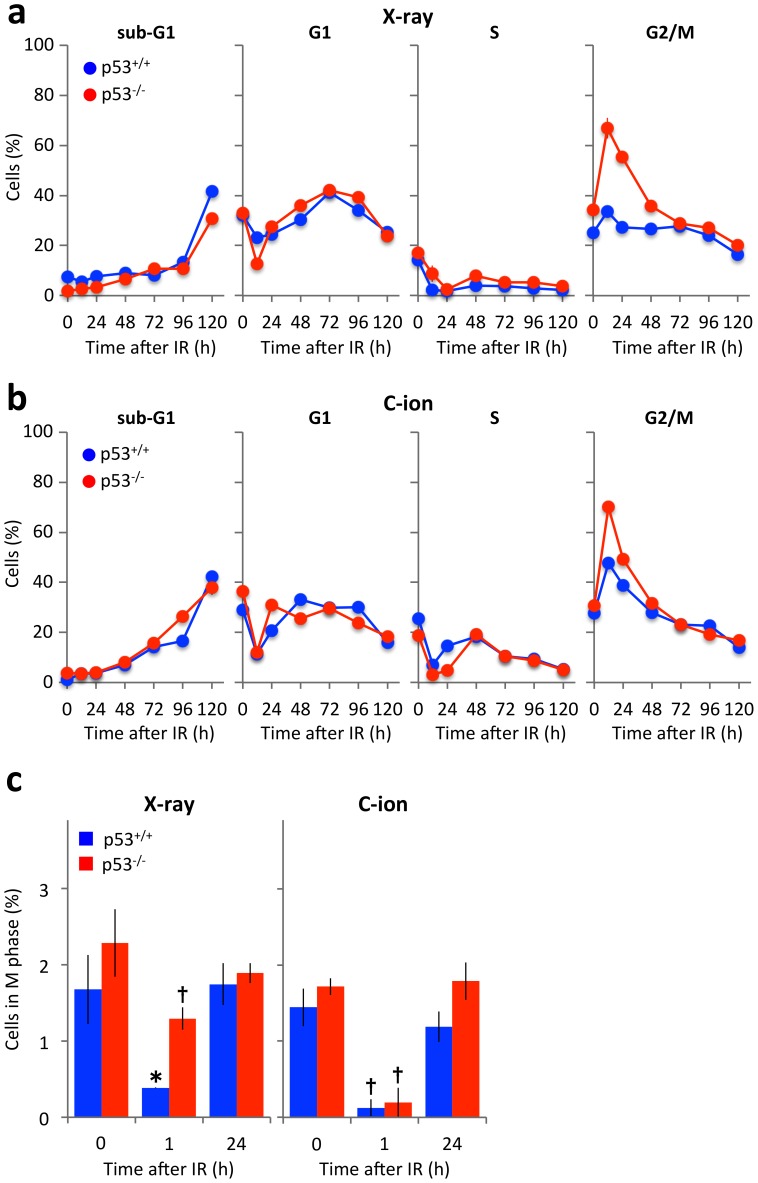
Cell cycle profiles of p53^+/+^ and p53^-/-^ HCT116 cells irradiated with X-rays or carbon-ion beams. Cells were seeded in 35 mm culture plates (a, b) or on glass coverslips (c), incubated overnight, and exposed (or not; 0 h) to X-ray (4 Gy) or carbon-ion beam (1.5 Gy) irradiation. (**a, b**) Cells irradiated with X-rays (a) or carbon-ion beams (b) were incubated for 0, 12, 24, 48, 72, 96 or 120 h, fixed with ethanol, stained with propidium iodide, and cell cycle status analyzed by flow cytometry. (**c**) Cells were irradiated with X-rays or carbon-ion beams, incubated for 1 h, and then subjected to immunostaining for pH3, a specific marker for M phase cells. Data are expressed as the mean ± SD. *P<0.05 and †*P*<0.01 versus the corresponding controls. IR, irradiation; C-ion, carbon-ion.

Mitotic catastrophe is thought to occur when cells proceed through aberrant mitosis with unrepaired DNA damage [26]. Therefore, to explore the mechanism underlying the induction of mitotic catastrophe in p53-null cells by carbon-ion beam irradiation, the effects of X-ray and carbon-ion beam irradiation on the cell cycle statuses of p53^+/+^ and p53^-/-^ HCT116 cells were determined by flow cytometry (**Fig. 6a, b**). Like the cell death analyses, the cells were irradiated with doses of X-ray (4 Gy) or carbon-ion beams (1.5 Gy). The induction of G2/M arrest that peaked 12 h after irradiation was observed in both cell lines after X-ray or carbon-ion beam irradiation, being more evident in the p53^-/-^ cells than p53^+/+^ cells. Notably, in both cell lines exposed to X-ray or carbon-ion beam irradiation, the G2/M arrest was fully released 48 h after irradiation.

Next, the percentages of p53^+/+^ and p53^-/-^ cells in the M phase before and after X-ray (4 Gy) or carbon-ion beams (1.5 Gy) irradiation were assessed by immunostaining using an antibody against pH3 (**Fig. 6c**) [24]. Approximately 2% of non-irradiated p53^+/+^ and p53^-/-^ cells were in the M phase. One hour after carbon-ion beam irradiation, the percentages of these cells in the M phase were reduced significantly, although p53^-/-^ cells were less susceptible than p53^+/+^ cells to X-ray irradiation. Notably, 24 h after X-ray or carbon-ion beam irradiation, the percentages of p53^+/+^ and p53^-/-^ cells in the M phase recovered to the baseline, suggesting that both cell lines restarted mitosis 24 h after the treatment.

### DNA double-strand breaks generated by carbon-ion beam irradiation show slower repair kinetics than those generated by X-ray irradiation

**Figure 7:**
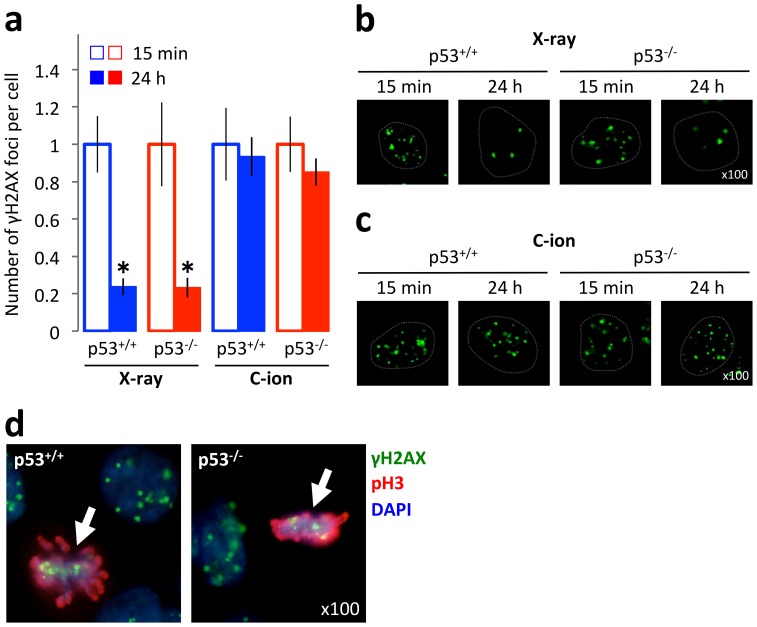
Kinetics of DNA double-strand break generation by X-ray or carbon-ion beam irradiation in p53^+/+^ and p53^-/-^ HCT116 cells. Cells were seeded on glass coverslips, incubated overnight, exposed to X-rays (2 Gy) or carbon-ion beams (1 Gy), incubated for an additional 15 min or 24 h, and then subjected to immunostaining for γH2AX and pH3. Cells were then stained with DAPI. (a) Numbers of γH2AX foci per cell at 15 min or 24 h post-irradiation. The results for each cell line were normalized to the number of γH2AX foci at the 15 min time point. At least 500 cells were counted per experimental condition. Data are expressed as the mean ± SD. *P<0.05 versus the corresponding samples at 15 min. (b, c) Representative microscopic images showing nuclei exposed to X-ray (b) or carbon-ion beam (c) irradiation, and immunostained for γH2AX. In each panel, the outline of the nucleus detected by DAPI staining is indicated by a dashed line. (d) Representative microscopic images of nuclei exposed to carbon-ion beam irradiation and immunostained for γH2AX and pH 3 at 24 h post-irradiation. The arrows indicate double-positive nuclei. C-ion, carbon-ion.

Finally, the repair kinetics of DNA double-strand breaks (DSBs), the most lethal type of DNA damage generated by ionizing irradiation, were examined in p53^+/+^ and p53^-/-^ HCT116 cells [27]. Irradiated cells were subjected to immunostaining using an antibody against γH2AX, and the numbers of γH2AX foci per cell at 15 min and 24 h post-irradiation were counted (**Fig. 7** **,** **S1 Table**) [24], [28]. The cells were irradiated with a 2 Gy dose of X-ray or a 1 Gy dose of carbon-ion beams; at these doses, the number of γH2AX foci per cell at the control time point (15 min post-irradiation) was approximately 20–30, which was appropriate for the assessment [24], [28]. Twenty four hours after X-ray irradiation, the numbers of γH2AX foci in p53^+/+^ and p53^-/-^ cells were 24±4.3% and 23±5.3% of those of the corresponding controls (at the 15 min time point), respectively (**Fig. 7a, b**), indicating that the large number of DSBs generated by X-ray irradiation were repaired within 24 h. By contrast, 24 h after carbon-ion beam irradiation, the numbers of γH2AX foci in p53^+/+^ and p53^-/-^ cells were 93 ± 11% and 85±7.3% of those of the corresponding controls, respectively (**Fig. 7a, c**), indicating that the DSBs generated by carbon-ion beam irradiation were not repaired efficiently, probably due to the structural complexity of DSB ends [29]. Indeed, p53^+/+^ and p53^-/-^ cells that stained double-positive for γH2AX and pH 3 were identified 24 h after carbon-ion beam irradiation, demonstrating that cells harboring DSBs had entered mitosis (**Fig. 7d**). The p53 status did not affect the kinetics of the loss of γH2AX foci after X-ray or carbon-ion beam irradiation. Taken together, these data suggest that p53-null cells harboring unrepaired DSBs enter mitosis 24 h after carbon-ion beam irradiation, leading to mitotic catastrophe.

## Discussion

Here, we demonstrate that carbon-ion beam irradiation induces distinct modes of cell death according to the mutation status of *TP53*. After both X-ray and carbon-ion beam irradiation, apoptosis was the dominant mode of cell death of p53^+/+^ cells but not p53^-/-^ cells. Notably, the rate of mitotic entry and the kinetics of DSB repair after irradiation, which may be key factors that induce mitotic catastrophe, were similar in p53^+/+^ and p53^-/-^ cells regardless of the type of irradiation used. These data indicate that apoptosis plays a primary role in cancer cell death caused by irradiation in the presence of p53. In the absence of p53, cancer cells showed resistance to apoptosis induction and mitotic catastrophe was observed after both X-ray and carbon-ion beam irradiation. This finding is likely explained by limitation of the G2/M checkpoint after irradiation. Activation of this checkpoint allows the repair of damaged DNA before it is passed on to daughter cells and acts as a barrier to prevent premature entry into mitosis [30]. However, previous studies have suggested the limitation of G2/M checkpoint after IR; G2/M checkpoint is released when the number of DSBs becomes lower than ∼10–20, followed by mitotic entry [24], [31]. Following the G2/M checkpoint release, cells harboring 10–20 DSBs are able to complete the mitotic event and enter the G1 phase [32], [33]. DSB repair is downregulated in the M phase; therefore, this damage may be repaired in the next cell cycle, although the repair process in daughter cells remains to be elucidated [34]. Another possible reason for the efficient induction of mitotic catastrophe in p53^-/-^ cells is the higher propensity of these cells to stall in the G2/M phase after irradiation than p53^+/+^ cells. This G2/M phase accumulation is the result of a defect in the p53-p21 signaling pathway that attenuates G1 arrest after irradiation [16]. This property of p53-deficient cancer cells might increase the chance of irradiated cells harboring unrepaired DSBs entering mitosis, leading to the enhancement of mitotic catastrophe.

The results of the present study suggest that both a lack of p53 and missense mutations in p53 contribute to the switch from apoptosis to mitotic catastrophe. Overall, 75% of the p53 mutations identified in human cancers are single missense mutations. Most missense mutations, including those examined in the present study, are located within the p53 DNA-binding domain, which plays a key role in the transcriptional activation of many target genes, including those that induce apoptosis [25]. Most mutant p53 proteins have a dominant-negative effect, leading to the dysfunction of the remaining normal p53 proteins. Therefore, it is reasonable that, along with the lack of p53, missense mutations in the p53 DNA-binding domain also contribute to the apoptosis-resistant phenotype by disrupting the ability of normal p53 proteins to transcriptionally activate apoptosis-related genes; this may render irradiated cells harboring unrepaired DSBs more susceptible to mitotic catastrophe. Nevertheless, it is worth noting a study limitation at this point: we were not able to establish H1299 cells expressing wild-type p53 (either transiently or stably); therefore, a comparison between wild-type p53 and mutant p53 was impossible. Future studies should compare the mode of irradiation-induced cell death in isogenic cell lines harboring wild-type, mutant, and null-p53.

Of note, the results presented here demonstrate efficient induction of mitotic catastrophe by carbon-ion beam irradiation in p53-null and p53-mutant cells. In fact, in all the p53-null and p53-mutant cells lines tested, the dose that are required to induce certain level of mitotic catastrophe was evidently lower in carbon-ion beams than in X-rays. This result can be explained by the difficulties associated with the repair of DSBs generated by carbon-ion beam irradiation, which retain more complex structures of damaged DNA ends than those generated by X-ray irradiation [35]. Inefficient DNA damage repair caused by the complexity of the DSB ends may underlie the efficient cell-killing effect of carbon-ion beam irradiation on cancer cells harboring p53 aberrations.

The results described here are partially contradictory to those of previous studies that examined the DDR after carbon-ion beam irradiation of p53-mutant cancer cells. Although a few studies observed efficient apoptosis (**S2 Table**) [12]–[15], it should be noticed that this mode of cell death was only induced efficiently at LET values greater than 70 keV/µm. By contrast, the average LET value at the center of the clinically-used spread-out Bragg peak, as used here, is approximately 50 keV/µm. In addition, in contrast to the results described here, the induction of senescence and prolonged (longer than 3 days) G2/M arrest was also observed in previous studies using carbon-ion beam irradiation with high LET values [12], [36]. These data suggest that the DDR differs depending on the LET value of the carbon-ion beam irradiation used. Additional *in vitro* and *in vivo* studies of a variety of cell lines are required to validate the therapeutic effects of carbon-ion beam irradiation at the LET used in clinical settings.

**Figure 8:**
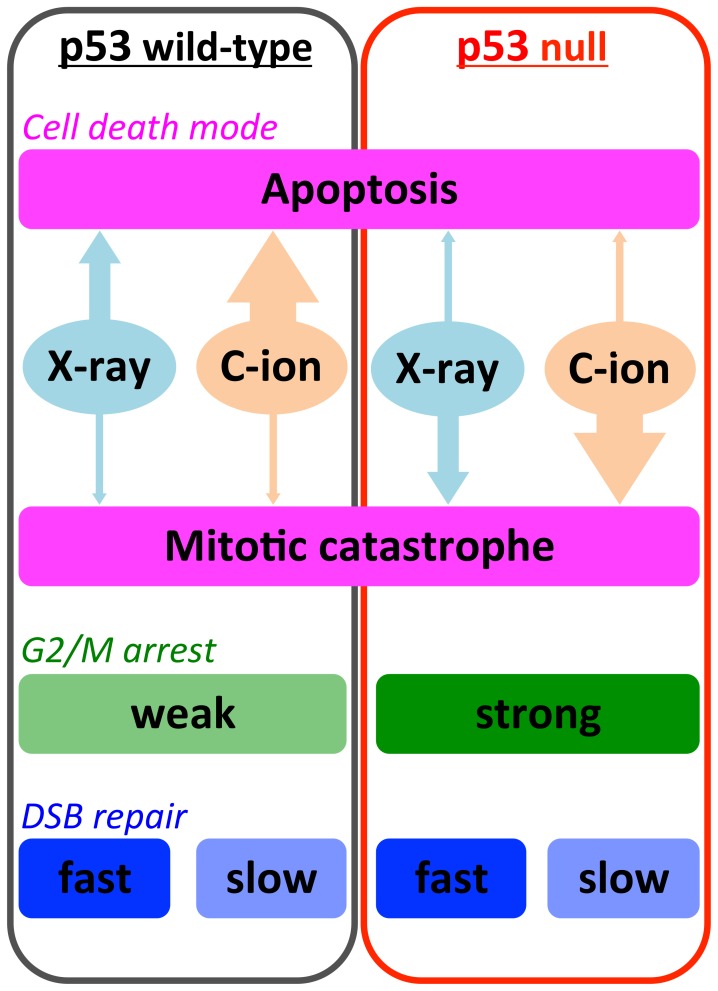
Schematic model outlining the DNA damage response and cell death modes in p53 wild-type and -null cells after X-ray or carbon-ion beam irradiation. C-ion, carbon-ion.

In summary, this comprehensive analysis of the DDR in irradiated isogenic cell lines demonstrates that X-ray irradiation-resistant p53-null cancer cells are susceptible to carbon-ion beam irradiation, which efficiently induces mitotic catastrophe (**Fig. 8**). The induction of mitotic catastrophe in apoptosis-resistant tumors may be an important biological advantage of carbon-ion radiotherapy over X-ray radiotherapy. Additional studies using animal models or clinical samples are required to elucidate this issue further.

## Supporting Information

S1 Fig **Properties of the p53^+/+^ and p53^-/-^ cells.**
(PDF)

S2 Fig **The modes of cell death induced by X-ray irradiation for the D_10_ in HCT116 p53^-/-^ cells.**
(PDF)

S3 Fig **The modes of cell death induced by X-ray or carbon-ion beam irradiation in BJ hTERT-WT or -shp53 cells.**
(PDF)

S1 Table **The number of γH2AX foci per cell after irradiation.**
(PDF)

S2 Table **LET-dependency of the efficacy of apoptosis induction by carbon-ion beam irradiation in p53-mutant cancer cells.**
(PDF)
