# Supplementary material for: Carbon-Ion Beam Irradiation Kills X-Ray-Resistant p53-Null Cancer Cells by Inducing Mitotic Catastrophe
Source: PLoS One. 2014 Dec 22;9(12):e115121. doi: 10.1371/journal.pone.0115121 (PMC4274003; doi:10.1371/journal.pone.0115121)
Supplement: S1 Fig — Properties of the p53+/+ and p53-/- cells. (PDF) [file pone.0115121.s001.pdf]

Supplementary Figure S1

a

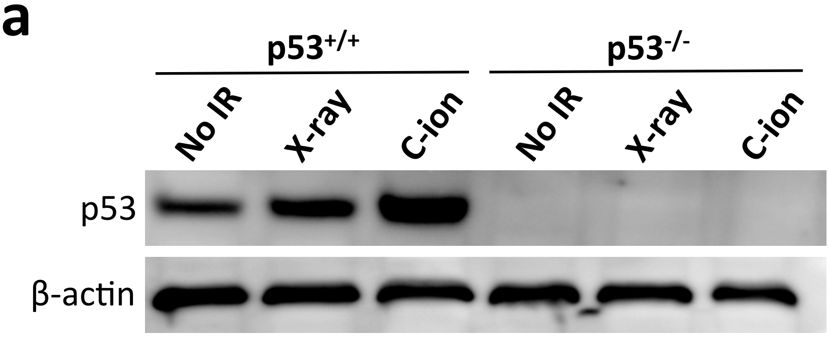

b

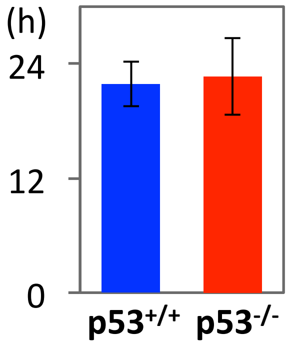

Supplementary Fig. S1. Properties of the $p53^{+/+}$ and $p53^{-/-}$ cells.
(**a**) Immunoblots showing X-ray (4 Gy) or carbon-ion beam (C-ion; 1.5 Gy) irradiation-induced expression of p53 in $p53^{+/+}$ cells, but not in $p53^{-/-}$ cells at 1 h post-irradiation. IR, irradiation.
(**b**) The population doubling times of the $p53^{+/+}$ and $p53^{-/-}$ cells. Cells were cultured in 100 mm dishes and passaged at a 5× dilution every 3 days. The number of cells was counted at the time of passage and the population doubling time calculated. Data are expressed as the mean ± SD.
