# Supplementary material for: Carbon-Ion Beam Irradiation Kills X-Ray-Resistant p53-Null Cancer Cells by Inducing Mitotic Catastrophe
Source: PLoS One. 2014 Dec 22;9(12):e115121. doi: 10.1371/journal.pone.0115121 (PMC4274003; doi:10.1371/journal.pone.0115121)
Supplement: S2 Fig — The modes of cell death induced by X-ray irradiation for the D10 in HCT116 p53-/- cells. (PDF) [file pone.0115121.s002.pdf]

Supplementary Figure S2

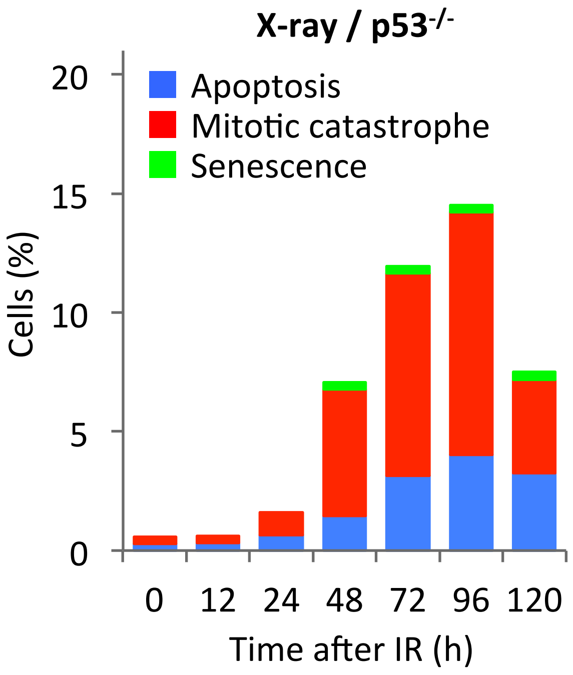

Supplementary Fig. S2. The modes of cell death induced by X-ray irradiation for the D_{10} in HCT116 $p53^{-/-}$ cells.

Cells were incubated overnight on coverslips and then irradiated (or not; 0 h) with 6.8 Gy X-rays. Cells were then stained with DAPI at the indicated times. Apoptosis, mitotic catastrophe, and senescence were determined according to the characteristic nuclear morphologies (see “Materials and methods” for the definitions). IR, irradiation.
