# Supplementary material for: Carbon-Ion Beam Irradiation Kills X-Ray-Resistant p53-Null Cancer Cells by Inducing Mitotic Catastrophe
Source: PLoS One. 2014 Dec 22;9(12):e115121. doi: 10.1371/journal.pone.0115121 (PMC4274003; doi:10.1371/journal.pone.0115121)
Supplement: S3 Fig — The modes of cell death induced by X-ray or carbon-ion beam irradiation in BJ hTERT-WT or -shp53 cells. (PDF) [file pone.0115121.s003.pdf]

Supplementary Figure S3

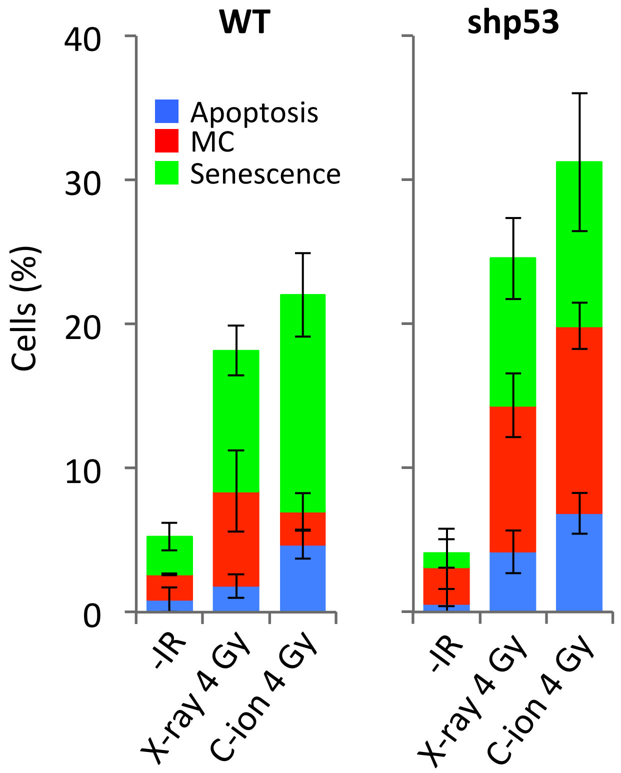

Supplementary Fig. S3. The modes of cell death induced by X-ray or carbon-ion beam irradiation in BJ hTERT-WT or -shp53 cells.
Cells seeded on coverslips, incubated for overnight, irradiated (or not) with X-rays (4 Gy) or carbon-ion beams (4 Gy), and then stained with DAPI 96 h later. Apoptosis, mitotic catastrophe, and senescence were determined according to the characteristic nuclear morphologies (see "Materials and methods" for the definitions). Data are expressed as the mean ± SD. MC, mitotic catastrophe; C-ion, carbon-ion; IR, irradiation.
