# Supplementary material for: Carbon-Ion Beam Irradiation Kills X-Ray-Resistant p53-Null Cancer Cells by Inducing Mitotic Catastrophe
Source: PLoS One. 2014 Dec 22;9(12):e115121. doi: 10.1371/journal.pone.0115121 (PMC4274003; doi:10.1371/journal.pone.0115121)
Supplement: S1 Table — The number of γH2AX foci per cell after irradiation. (PDF) [file pone.0115121.s004.pdf]

Supplementary Table S1

The number (mean ± SD) of γH2AX foci per cell at the indicated times after irradiation.

		15 min	24 h
X-rays (2 Gy)	p53+/+	34.6 ± 5.2	8.2 ± 0.5
	p53-/-	30.3 ± 6.8	6.8 ± 0.3
Carbon-ion (1 Gy)	p53+/+	21.6 ± 4.2	20.2 ± 1.6
	p53-/-	22.9 ± 3.4	18.7 ± 1.4
