# Supplementary material for: Carbon-Ion Beam Irradiation Kills X-Ray-Resistant p53-Null Cancer Cells by Inducing Mitotic Catastrophe
Source: PLoS One. 2014 Dec 22;9(12):e115121. doi: 10.1371/journal.pone.0115121 (PMC4274003; doi:10.1371/journal.pone.0115121)
Supplement: S2 Table — LET-dependency of the efficacy of apoptosis induction by carbon-ion beam irradiation in p53-mutant cancer cells. (PDF) [file pone.0115121.s005.pdf]

Supplementary Table S2

LET-dependency of the efficacy of apoptosis induction by carbon-ion beam irradiation in p53-mutant cancer cells.

Efficient apoptosis induction*	LET (keV/μm)	Dose (Gy)	Cell line	Endpoint	References
Yes	184	3, 5	SQ20B	sub-G1 fraction	12
Yes	100	1	SAS	DNA fragmentation etc.	15
Yes	100, 70	2	Ca9-22	PARP and caspase-3 cleavage	13
Yes	70	~3 (D30†)	H1299	Hoechst 33342 staining	14
No	70, 30	1	SAS	DNA fragmentation etc.	15
No	33.6	3, 5	SQ20B	sub-G1 fraction	12
No	30, 13	2	Ca9-22	PARP and caspase-3 cleavage	13

*"Yes" indicates that carbon-ion beam irradiation was a better inducer of apoptosis than X-ray irradiation at the iso-survival dose. †D30, the dose at which 30% of cells survived.
